# Cutaneous Leishmaniasis in an Immunocompromised Pediatric Patient With Acute Lymphoblastic Leukemia

**DOI:** 10.7759/cureus.14203

**Published:** 2021-03-31

**Authors:** Stephanie R Cohen, Carolina Espinoza, Kathia Valverde Muñoz

**Affiliations:** 1 Dermatology, Tufts Medical Center, Boston, USA; 2 Internal Medicine, Hospital San Juan de Dios, San José, CRI; 3 Hematology, Hospital Nacional de Niños, San José, CRI

**Keywords:** cutaneous leishmaniasis, neglected tropical disease, acute lymphoblastic leukemia, immunocompromised

## Abstract

Leishmaniasis is a protozoan disease caused by the parasite Leishmania. It is most common in developing countries. Its clinical presentation varies depending on several factors such as patient’s immunity. Cutaneous leishmaniasis is one of the main types of leishmaniasis, it is known to be a great mimicker. When seen in immunodeficient populations, such as patients with acute lymphoblastic leukemia, it may present more aggressively and its diagnosis is challenging. We present a case of a five-year-old male with a history of acute lymphoblastic leukemia undergoing chemotherapy who developed papules evolving into ulcerated nodules on his left lower extremity. An initial smear for leishmaniasis was negative, the disease evolved and spread in an ascending fashion, while efforts were made finding a diagnosis. One-month later the smear was repeated and positive for leishmaniasis. Subsequently, therapy with Meglumine antimoniate was prescribed. The lesions healed with atrophic scarring without complications. Cutaneous leishmaniasis diagnostic methods are not standardized, limitations such as interpreter’s expertise and patient’s immunity state may play a role in delaying the diagnosis.

## Introduction

Leishmaniasis is an infectious, protozoan disease caused by the parasite Leishmania. It is transmitted to humans by the phlebotomine sandfly bite (Lutzomyia ylephiletor and Lutzomyia trapidoi are the two found in Costa Rica) and it is endemic to developing regions such as Central and South America, the Middle East, and India [1]. The three main clinical manifestations are cutaneous, mucocutaneous, and visceral. The presentation depends on the parasite genus, geographic area, and patient’s immunity.

Acute lymphoblastic leukemia (ALL) is the most common malignancy in the pediatric population. ALL and the chemotherapy treatment induce a state of immunodeficiency which allows for opportunistic infections [2]. We report a case of disseminated cutaneous leishmaniasis (CL) presenting in a pediatric patient with ALL undergoing maintenance phase chemotherapy. This article was previously presented as a poster in the 24th World Congress of Dermatology Milan 2019.

## Case presentation

**Figure 1:**
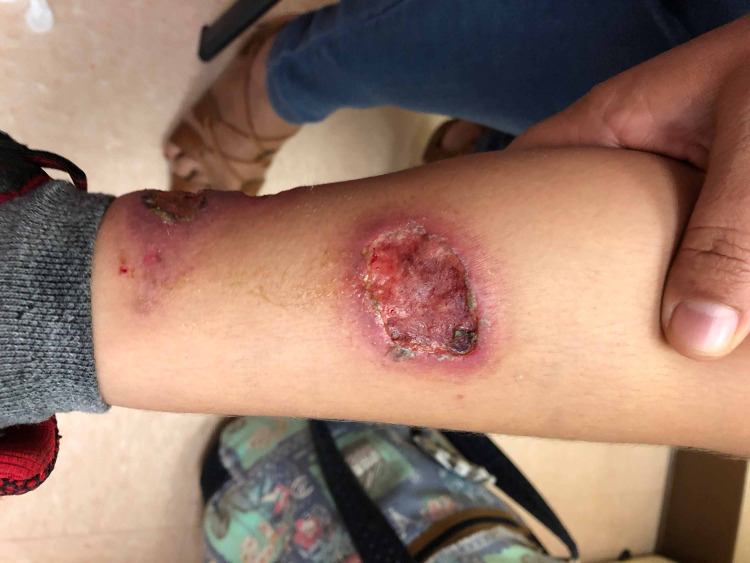
Ulcerated nodules pre-treatment. Multiple ulcerated erythematous-violaceous nodules with raised crusted borders on the left lower limb.

A five-year-old male with a history of ALL undergoing CRI-16 chemotherapy regimen (dexamethasone, vincristine, methotrexate and 6-mercaptopurine) in maintenance phase-cycle 2 presented to Costa Rica’s National Children’s Hospital (Hospital Nacional de Niños Dr. Carlos Sáenz Herrera) with a one-month history of an erythematous papule in the left lower limb that evolved into multiple ulcerated nodules (Figure 1).

**Figure 2:**
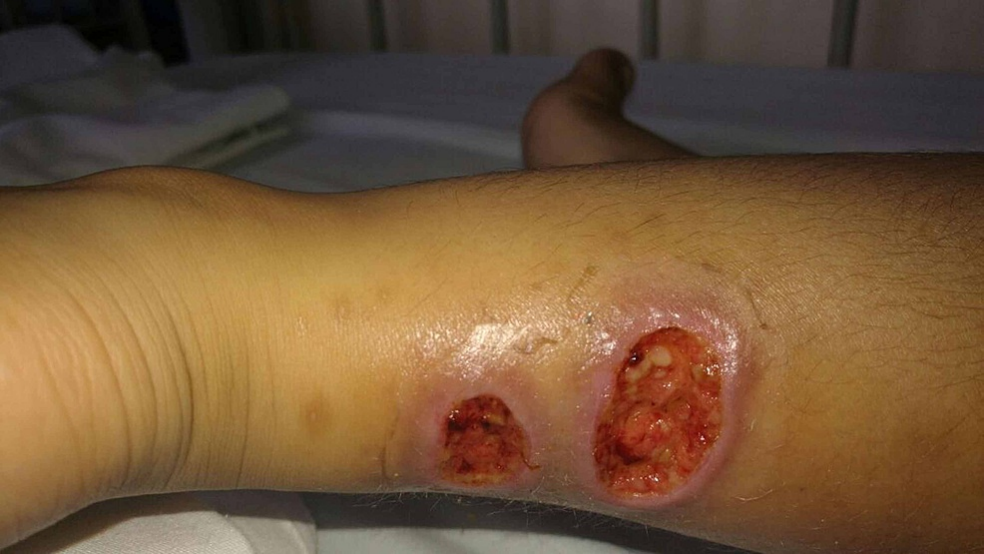
Ulcers after one month of antibiotic. Multiple erythematous ulcers with moist based and raised borders on the left lower limb.

As per the patient’s mom, the primary lesions developed secondary to a mosquito bite. A smear for leishmania was done at a regional hospital with negative results. At the moment, the patient was treated with topical fusidic acid and trimethoprim/sulfamethoxazole for one month with no improvement and ascending spread of the lesions. One month after the first consult, physical exam was remarkable for three ulcerated nodules with erythemato-violaceous borders over the left lower limb and crusted scaly plaques on the right buttock, with multiple adenopathies in an ascending fashion (Figure 2).

**Figure 3:**
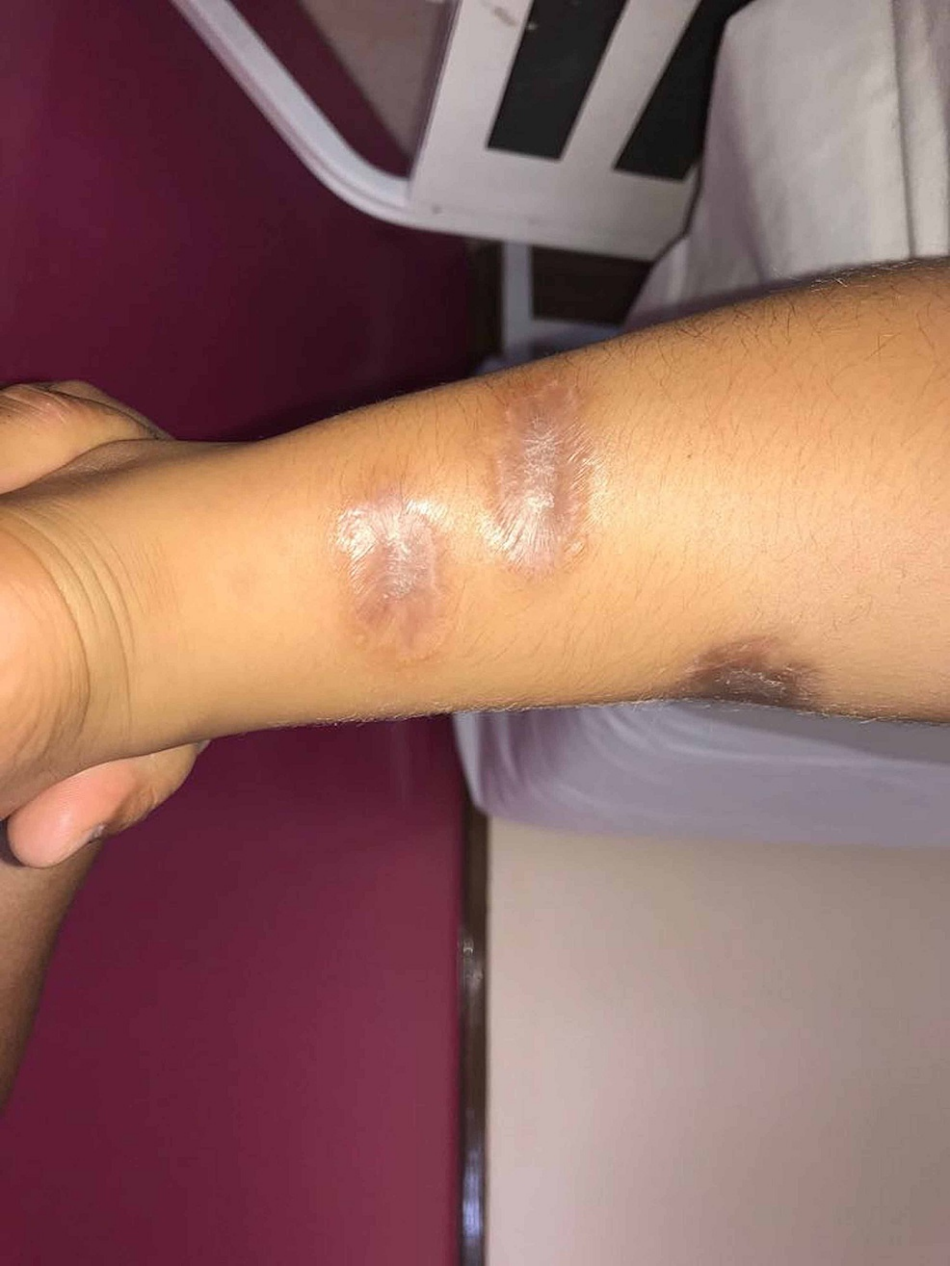
Post treatment. Retracted hypopigmented atrophic scarring over the left lower limb.

A new smear was done and results were positive for leishmaniasis. Treatment with intramuscular Meglumine antimoniate was prescribed at 1250mg/day (56mg/kg/day) for 21 days, without any side effects. At this moment absolute neutrophil count was 1620/uL and total leucocyte count 3030/uL. Chemotherapy was suspended during the time of treatment. The lesions healed with atrophic scarring (Figure 3).

Upon completion of treatment, the patient was able to restart chemotherapy without complications. At the time of this publication the patient is a cancer survivor, being one year off treatment.

## Discussion

CL is a zoonotic parasitic infection transmitted by the female phlebotomine sandfly. Two species (Lutzomyia ylephiletor and Lutzomyia trapidoi) and three reservoirs (three-toed sloths [Bradypus sp and Choloepus sp] and rodents [Heteromys desmarestianus]) are found in Costa Rica. It is considered a neglected tropical disease with an incidence of approximately two million cases per year [3]. Costa Rica has an average annual incidence rate of this parasitic infection of 20.3 per 100,000 people [4].

CL’s clinical presentation may mimic malignancies such as cutaneous basal and squamous cell carcinoma, and other infections such as syphilis, sporotrichosis, and ecthyma. When diffuse it may resemble conditions such as lepra, lobomycosis, and lupus vulgaris [1]. Given CL’s kinship with a wide variety of diseases, clinicians must have a high level of suspicion to diagnose it. Moreover, its diagnosis is challenging due to a lack of test standardization [5].

Even though, CL infections may resolve clinically without treatment, and not all patients who undergo treatment demonstrate elimination of parasitic infection, an accurate and prompt diagnosis is key to prevent complications. Further, some diagnostic tests are operator-dependent and access to some methods may be limited in developing regions. Polymerase chain reaction (PCR) seems to be the most sensitive and specific diagnostic tool [6]. Other methods such as biopsies, cultures, smears, and dermal scrapings are accepted, however, even when performed by experts only in 70% of CL cases the parasite is identified. The Leishmanin (Montenegro) skin test does not differentiate between past and present infection and may elicit a false negative report in diffuse CL and immunosuppressed patients [1].

The presented patient had a classic CL presentation and lived in a region in northern Costa Rica where leishmaniasis is endemic [4]. Because of this, CL suspicion was high in the differential diagnosis. However, therapy was delayed due to an initial false negative smear (sensitivity 54%) [6], causing a typical clinical presentation of CL at the time of diagnosis.

Multiple factors can contribute to false-negative results such as the immunocompromised state of the patient (due to his chemotherapy regimen), area of the lesion where the smear was obtained, time from inoculation, and interpreter’s expertise. It is not recommended to treat CL empirically since therapy is prolonged and toxic. In immunosuppressed patients CL may be more aggressive and disseminated [1].

Limitation to our study includes not being able to follow the evolution of the disease from a single to three lesions since the patient presented to our institution later on when the CL had already spread.

## Conclusions

CL’s ability to mimic other diseases, especially in immunocompromised patients, makes its diagnosis a challenge. Perhaps because of the complexity of their underlying disease added to the fact that leishmaniasis diagnostic methods are convoluted. Regardless, maintaining a high level of suspicion for CL in endemic regions, especially if associated with immunodeficiency states, is of utmost importance for the diagnosis of this disease and avoiding the relapsing tendency and severe atrophic scarring in those not treated in a timely manner. In rural areas there is no access to molecular diagnostic methods, only access to smear analysis is available. Nowadays molecular methods are available at the National Children’s Hospital in San José.
